# Risk of anaphylaxis in patients with large local reactions to hymenoptera stings: a retrospective and prospective study

**DOI:** 10.1186/s12948-015-0030-z

**Published:** 2015-11-09

**Authors:** Stefano Pucci, Simona D’Alò, Tiziana De Pasquale, Ilenia Illuminati, Elena Makri, Cristoforo Incorvaia

**Affiliations:** Allergy Unit, General Hospital, Civitanova Marche, Italy; Allergy/Pulmonary Rehabilitation, ICP Hospital, via Bignami 1, 20126 Milan, Italy

**Keywords:** Hymenoptera sting, Large local reaction, Systemic reaction, Retrospective, Prospective

## Abstract

**Background:**

In the few studies available, the risk of developing systemic reactions (SR) to hymenoptera stings in patients with previous large local reactions (LLRs) to stings ranges from 0 to 7 %. We evaluated both retrospectively and prospectively the risk of SRs in patients with LLRs to stings.

**Methods:**

An overall number of 477 patients, 396 with an SR as the first manifestation of allergy and 81 with a history of only LLRs after hymenoptera stings, were included in the study. All patients had clinical history and allergy testing (skin tests and/or specific IgE) indicative of allergy to venom of only one kind of Hymenoptera. Of the 81 patient with LLRs, 53 were followed-up for 3 years by annual control visits, while the 396 patients with SR were evaluated retrospectively.

**Results:**

Among the 396 patients with an SR, only 17 (4.2 %) had had a previous LLR as debut of allergy, after an history of normal local reactions to Hymenoptera stings. All the 81 patients with a history of only LLRs had previously had at least two LLRs, with an overall number of 238 stings and no SR. Among the 53 patients who were prospectively evaluated we found that 31 of them (58.3 %) were restung by the same type of insect, with an overall number of 59 stings, presenting only LLRs and no SR.

**Conclusions:**

Our findings confirm that patients with repeated LLRs to stings had no risk of SR, while a single LLR does not exclude such risk. This has to be considered in the management of patients with LLRs.

## Background

Large local reactions (LLRs) to Hymenoptera stings, that are defined as skin reactions around the site of the sting characterized by edema, erythema and itching, with a diameter greater than 10 cm, are much more common than systemic reactions (SRs) [1]. In fact, prevalence of LLRs as high as 26.4 and 38 % were reported in the general population [2] and in beekeepers [3], respectively, compared with a mean prevalence of SRs to stings of around 3 % in adults and 1 % in children [4]. The most relevant medical aspect of LLRs is the natural history, and in particular the risk of developing an SR to a further sting of the same kind of insect. The first studies on this issue date back to 1984, when Graft et al. [5] and Mauriello et al. [6] published their observations on 54 children and 18 adults, respectively. The rate of SR following a history of LLRs detected in these studies was very similar, corresponding to 4 % in children [5] and 5 % in adults [6]. By these results, the authors concluded that patients with LLRs tend to repeat such reactions, and that the risk of anaphylaxis is too small to warrant venom immunotherapy. Further studies were published only 20 years later. In 2004, Golden et al. [7] reported in 44 children with LLRs a 7 % rate of SRs, while in 2005 a survey from Spain showed that no SR to subsequent stings was developed by 23 patients with LLRs during a 4-year follow-up [8]. In 2009, a review on the issue suggested that further studies were needed on the natural history of LLRs, also based on authors’ personal observation of a risk for SRs in patients with LLRs higher than reported [1].

 We aimed this study at evaluating, both retrospectively and prospectively, the risk of SRs subsequent to LLRs in a large population of patients with Hymenoptera venom allergy.

## Patients and methods

An overall number of 477 patients, 396 who had had an SR as the first manifestation of allergy and 81 with a history of only LLRs after hymenoptera stings, were included in the study. All patients had clinical history and allergy testing (skin tests and/or specific IgE) indicative of allergy to venom of only one kind of Hymenoptera. The history data disclosed the presence of atopy in 12.3 % of patients with LLRs and 10.1 % of patients with previous SRs. There was no correlation between the kind of reaction and the site of stings. There were 4 beekeepers in the 81 patients with only LLRs (4.9 %) and 15 beekeepers in the 396 patients with previous SRs (3.8 %), this difference being not significant. Four patients with previous SRs had had a diagnosis of mastocytosis based on high levels of tryptase and bone marrow biopsy. No patient had a positive history for other immunological diseases. The patients were instructed on how to recognize the different types of Hymenoptera according to morphological characteristics. This criterion was chosen to avoid confusing data concerning the field stings reported by the patients. Of the 81 patient with LLRs, 53 were followed-up for 3 years by annual control visits, while the 396 patients with SR were evaluated retrospectively. The data were obtained through standard practice not requiring ethics approval. However, the local Ethical Committee was informed about the procedure of the study, and patients gave their consent to be included in the study. Table 1 reports the demographic data and the responsible insects in the two groups of patients.

**Table 1:** Characteristics of the two groups of patients

	Males/females	Mean age (years)	Age range (years)	Culprit insect
Group 1 (retrospective, SRs) 396 patients	298/98	46.8	8–86	180 *Vespula spp*. 114 *Polistes spp*. 36 *Vespa crabro* 66 *Apis mellifera*
Group 2 (prospective, LLRs) 81 patients	58/23	38.5	8–77	38 *Vespula spp*. 33 *Polistes spp.* 10 *Apis mellifera*

## Results

Among the 396 patients with an SR, only 17 (4.2 %) had had a previous LLR as the first manifestation of allergy, after an history of normal local reactions to insect stings. According to the Mueller classification of severity of SR to insect stings [9] 8 patients had a grade I, 64 patients a grade II, 155 a grade III, and 169 a grade IV SR. All the 81 patients with a history of only LLRs had previously had at least two LLRs, with an overall number of 238 stings (corresponding to a mean number of 2.94 stings for each subject, range 1–10), and no SR. The time of onset of the LLR ranged between 1 and 8 h. Of the 81 patients with LLRs, in the 53 patients who were prospectively evaluated we found that 31 of them (58.3 %) were restung by the same type of insect, with an overall number of 59 stings (corresponding to a mean number of 1.90 stings for each subject, range 1–6), presenting only LLRs; no patient had an SR (Table 2).

**Table 2:** Results of the prospective part of the study

No. of patients with LLR	Patients followed for 3 years	Patient restung	No of LLR (pts)	No of SR
81	53	31	59 (31)	0

## Discussion

LLRs to Hymenoptera stings may be IgE-mediated or not. Green et al. in 1980 reported that, in 22 patients who had LLRs from Hymenoptera stings, approximately half of them had venom-specific IgE antibodies, and that no correlations could be found between the presence of venom-specific IgE and age, sex, sting location, atopic history, or prior stings [10]. This suggested to the authors that after an LLR from an insect sting patients must be individually assessed for the presence of venom-specific IgE and considered for specific immunotherapy. Subsequent studies focused the interest on IgE-mediated LLRs and particularly on the risk to develop SRs after an LLR. Four studies on this issue are available thus far. The first two studies have similar results, with a rate of SRs of 4 % in children [5] and 5 % in adults [6]. Instead, contrasting data were reported in the two more recent prospective studies, with a rate of SRs of 7 % in children [7] but no SR at all in a group of patients including both children and adults [8]. The major issues concerning these studies are the known limitations of retrospective studies and the low number of patients in the prospective studies. Concerning the first issue, the biases which can negatively impact the reliability of this type of study include the selection bias, the difficulty in assessing the temporal relationship and control of outcomes, and the need of large sample size for rare outcomes [11]. In the case of allergic reactions to insect stings, the psychological characteristics of patients may influence their decision to undergo a medical evaluation, being possible that patients claim such evaluation after an LLR or feel it unnecessary after an SR. These bias weaken the findings of retrospective studies, while the prospective evaluation allows more reliable observations. Still, a small number of patients makes it unlikely for statistical reasons to achieve a sound consistency also for prospective studies. In particular, the contrasting outcome of the two prospective studies in patients with LLRs—7 vs. 0 %—were observed in two groups of 44 children and 23 children and adults, respectively. In the prospective part of our study we included 81 adult patients with at least two LLRs, but only 53 were evaluated for 3 years, by annual control visits, 31 of them being re-stung with no SR, that confirms the finding by Fernandez et al. [8]. Concerning the retrospective part of the study, in the 396 patients with an SR only 4.2 % had had a previous single LLR as debut of allergy, that is the identical rate observed in the study by Mauriello et al. [6] in 118 patients with SR.

The most interesting data of our study is the correlation between the number of LLRs and the risk to develop an SR. In fact, we observed that in patients with a single LLR as the first manifestation of venom allergy there is a risk, although low, of SR to a subsequent hymenoptera sting, while there is no risk of SR in presence of at least two previous consecutive LLRs.

These findings may be useful in practical management of patients sensitized to hymenoptera venom, especially concerning the prescription of epinephrine auto-injectors. Actually, patients with at least two LLRs to stings are unlikely to need an auto-injector because there is no apparent risk of SR, while in patients evaluated after a single LLR the risk of SR cannot be ruled out and the availability of epinephrine for auto-injection is worthwhile. However, only a prospective study with a prolonged follow-up on a large number of patients with LLRs to stings could definitely settle the issue.
